# Health System Resource Gaps and Associated Mortality from Pandemic Influenza across Six Asian Territories

**DOI:** 10.1371/journal.pone.0031800

**Published:** 2012-02-21

**Authors:** James W. Rudge, Piya Hanvoravongchai, Ralf Krumkamp, Irwin Chavez, Wiku Adisasmito, Pham Ngoc Chau, Bounlay Phommasak, Weerasak Putthasri, Chin-Shui Shih, Mart Stein, Aura Timen, Sok Touch, Ralf Reintjes, Richard Coker, on behalf of the AsiaFluCap Project Consortium

**Affiliations:** 1 Communicable Diseases Policy Research Group, London School of Hygiene and Tropical Medicine, Bangkok, Thailand; 2 Department of Health Sciences, Hamburg University of Applied Sciences, Hamburg, Germany; 3 Faculty of Tropical Medicine, Mahidol University, Bangkok, Thailand; 4 Faculty of Public Health, University of Indonesia, Depok, Indeonesia; 5 Vietnam Military Medical University, Hanoi, Vietnam; 6 National Emerging Infectious Diseases Coordination Office, Ministry of Health, Vientiane, Lao People's Democratic Republic; 7 International Health Policy Programme-Thailand, Nonthaburi, Thailand; 8 Taiwan Centre for Disease Control, Taipei, Taiwan; 9 National Institute for Public Health and the Environment (RIVM), Bilthoven, The Netherlands; 10 Department of Communicable Disease Control, Ministry of Health, Phnom Penh, Cambodia; University of Hong Kong, Hong Kong

## Abstract

**Background:**

Southeast Asia has been the focus of considerable investment in pandemic influenza preparedness. Given the wide variation in socio-economic conditions, health system capacity across the region is likely to impact to varying degrees on pandemic mitigation operations. We aimed to estimate and compare the resource gaps, and potential mortalities associated with those gaps, for responding to pandemic influenza within and between six territories in Asia.

**Methods and Findings:**

We collected health system resource data from Cambodia, Indonesia (Jakarta and Bali), Lao PDR, Taiwan, Thailand and Vietnam. We applied a mathematical transmission model to simulate a “mild-to-moderate” pandemic influenza scenario to estimate resource needs, gaps, and attributable mortalities at province level within each territory. The results show that wide variations exist in resource capacities between and within the six territories, with substantial mortalities predicted as a result of resource gaps (referred to here as “avoidable” mortalities), particularly in poorer areas. Severe nationwide shortages of mechanical ventilators were estimated to be a major cause of avoidable mortalities in all territories except Taiwan. Other resources (oseltamivir, hospital beds and human resources) are inequitably distributed within countries. Estimates of resource gaps and avoidable mortalities were highly sensitive to model parameters defining the transmissibility and clinical severity of the pandemic scenario. However, geographic patterns observed within and across territories remained similar for the range of parameter values explored.

**Conclusions:**

The findings have important implications for where (both geographically and in terms of which resource types) investment is most needed, and the potential impact of resource mobilization for mitigating the disease burden of an influenza pandemic. Effective mobilization of resources across administrative boundaries could go some way towards minimizing avoidable deaths.

## Introduction

Recent experience from the 2009-H1N1 pandemic highlights how health system capacities, even in developed countries, can be stretched by relatively mild pandemic scenarios [1]–[3]. Indeed, the vast majority of previous health system analyses in relation to pandemic influenza have focused on developed countries [1], [3]–[11], while the capacity of health systems in low and middle income countries remains largely unstudied. Paradoxically, understanding outbreak response capacity in low and middle-income countries is arguably of greater importance than that in developed countries, not only because health systems are weaker [12], but also because many of these countries are in regions where the risk of emerging infectious diseases is highest [13]. Moreover, these countries may suffer disproportionately because of associations between morbidity, pandemic influenza and poverty [14].

Proposed strategies for pandemic preparedness in many countries frequently focus on development and acquisition of pandemic vaccines and stockpiling and distribution of antiviral drugs [15]–[16]. In the Southeast Asia region, while surveillance and outbreak response capacities have been strengthened in hope of early detection and control of outbreaks, there has been much less investment into preparing health systems for pandemic mitigation [17]. Modeling studies have been used to inform optimum intervention strategies for responding to pandemic influenza, but often neglect to take into account feasibility of health systems to implement such a response and the potential impact of resource shortages on the pandemic burden.

Investigation of health system capacity in East and Southeast Asia is of particular interest, not only given the fertile conditions for the emergence and spread of new diseases [13], [18], but also the wide socio-economic inequalities within the region, and focus of investment by the international community into pandemic influenza preparedness [19]. Resource gaps for a pandemic response are likely to be wide and vary greatly between and within countries in Asia [20]. But exactly how wide are these gaps, what are the consequences of the gaps in terms of the pandemic disease burden, and to what extent could these consequences be mitigated by improving resource allocation and mobilization?

To address these questions, we conducted a health systems analysis across six Asian countries and territories with widely varying socioeconomic conditions: Cambodia, Indonesia, Lao PDR, Taiwan, Thailand and Vietnam. In this analysis, mathematical modeling and health system resource data collected across the six territories were used to estimate and compare, within and across countries, the resource gaps, and potential consequences of those gaps in terms of expected mortalities, for a hypothetical pandemic influenza scenario.

## Methods

This study was conducted as part of the *AsiaFluCap* project (www.asiaflucap.org), the overall aim of which is to conduct health systems analyses to support capacity development for responding to pandemic influenza across six countries and territories in Asia, specifically: Cambodia, Indonesia, Lao PDR, Taiwan, Thailand and Vietnam.

### Health system resource data

For this comparative analysis we focus on four key health system resources: antiviral drugs (specifically oseltamivir), hospital beds, mechanical ventilators and healthcare workers (doctors and nurses), chosen due to their critical importance for responding to pandemic influenza. These resources, along with over 50 other resource items relating to health system infrastructure, equipment, materials, and human resources, were selected through a systematic literature review and a Delphi consensus process by a panel of 24 experts, as described in [21]. Quantities of these resource items were enumerated during March to September 2009 through questionnaires administered to hospitals and health offices in all districts of each of the six study countries (except Indonesia, where data were collected only from districts in Jakarta and Bali due to the vast geographic scale of the country). Additional questionnaires were sent to ministries of health to capture central stockpiles.

We received 100% response rates from hospitals and district health offices in Cambodia. Overall response rates from district health offices were more than 95% in Viet Nam and Indonesia (Jakarta and Bali), 86% in Lao PDR, 72% in Taiwan, and 59% in Thailand. The response rates for hospital questionnaires were slightly lower at 93% for Indonesia and Vietnam, 70% for Lao PDR, and approximately 46% for Thailand and Taiwan. Data from the questionnaires were double-entered into an Excel database. Missing values, due to non-responses or incomplete questionnaires, were extrapolated using linear prediction models, specific to each country and resource item, based on a number of district characteristics such as total number of hospital beds or public hospital beds, population size, and geographic location (region/province). For oseltamivir and ventilators, two-step models were used to first estimate the likelihood of having any oseltamivir or ventilators, and then to predict the number of these items. Extrapolation of missing data was carried out in STATA version 11.

### Epidemic modeling and links to health systems resources

In order to estimate health system resource needs and gaps for a pandemic influenza scenario, we used a mathematical model previously developed as part of the AsiaFluCap project to simulate the transmission dynamics of a pandemic influenza outbreak [22]. Full details and equations of the model can be found in [22], and are summarized in Text S1. Briefly, the model is based on a deterministic SEIR (Susceptible-Exposed-Infectious-Recovered/removed) model described by differential equations tracking number of people in each compartment over time. Given that the primary aim of this analysis was to provide relative estimates of resource gaps within and across countries, rather than to accurately simulate the spread of pandemic influenza throughout each country, the model structure was kept relatively simple, with homogeneous mixing patterns and no age-structure assumed within the modeled population. However, novel complexity is incorporated through making parameters describing the clinical course of infected individuals conditional upon the availability of certain key health system resources (antivirals, beds, and ventilators). Thus we could obtain relative indications of the consequences of resource shortages on the pandemic disease burden, specifically in terms of “avoidable deaths”, which we define as deaths that would not have occurred in the presence of sufficient resources.

The infectious compartment of the model was subdivided into three groups based on clinical severity: asymptomatic, mild and severe infections. All asymptomatically and mildly infected patients were assumed to recover, while severe cases were at risk of death and were assumed to need antiviral treatment and hospitalized care, although whether they received either of these depended upon the availability of oseltamivir and hospital beds, respectively. Treatment with antivirals and hospitalization were assumed to reduce the infectious period and the probability of death for severe cases. Furthermore, a proportion of severe cases are assumed to require mechanical ventilation, which they will receive as long as ventilators are available, otherwise these cases would die.

The model parameters describing transmissibility and clinical severity were chosen based on data from the 2009-H1N1 pandemic, with a basic reproduction number of 1.32 [23]–[24]. Under the parameter values chosen (see Table S1 and [22]), in a population with sufficient resources (i.e. when there are no shortages of oseltamivir, hospital beds, or ventilators), the scenario predicts an overall attack rate of 35.6%, a clinical attack rate of 24.9%, a peak prevalence (of symptomatic cases) of 0.94%, and a case fatality rate of 0.018%. In the absence of any resources, the case fatality rate is substantially higher at 0.029%, while attack rates and peak prevalence remain very similar.

### Estimating resource needs, gaps, and associated mortality

In our baseline scenario, resource gaps were estimated assuming that 12% of “general” hospital resources (beds, ventilators and human resources) are available for care of pandemic influenza cases, with the remaining 88% required for maintaining essential healthcare services, as in a previous pilot study for Thailand [20], and based on previous reports [25]–[26]. We also assumed that, in the event of a pandemic all available oseltamivir doses would be dedicated to severe influenza cases. These assumptions regarding resource spare capacity and oseltamivir usage were relaxed in a multivariate uncertainty analysis (described below). The resource data were aggregated at provincial level for all countries except Taiwan, where they were aggregated at county level, and Indonesia, where the data were kept at district level. (Counties in Taiwan and districts in Indonesia are of comparable population size to the provinces of the other four countries.) We then ran the model separately for each province (Lao PDR, Cambodia, Thailand, Vietnam), county (Taiwan) and district (Indonesia), with the appropriate resources for each of these administrative areas, assuming a closed population and that resources could not be shared between these areas in a timely manner. For the purposes of narrative flow, we henceforth use the term “province” for counties in Taiwan. All simulations started with one mild case entering a completely susceptible population.

In addition to running the model with the available resources, based on the survey data, we also ran the model with unlimited resources, in order to calculate resource needs, and thus also the resource gaps (or indeed surpluses) by comparing with the resource availability data. The needed number of hospital beds, ventilators and humans resources were estimated from the peak number of cases requiring hospitalization and ventilation, while the needed number of oseltamivir doses was calculated from total number of severe cases occurring over the duration of the outbreak (full details on assumptions of resource depletion rates are detailed in [22], and summarized in Text S1). By comparing the number of deaths predicted by simulations with sufficient resources with those from simulations using actual resource data, we also estimated the number of deaths due to resource gaps, which we term as “avoidable deaths”.

A multivariate uncertainty analysis was conducted to approximate uncertainty surrounding avoidable deaths in light of uncertainty in resource spare capacity and effectiveness, which may vary between settings. Specifically, the proportion of “general” healthcare resources within each province/district that would be available to care for pandemic influenza patients was allowed to vary between 5–20%, and parameters describing the effectiveness of each resource for reducing the risk of death in cases requiring those resources were allowed to vary independently between a wide range of 20–80%. We also explored the impact of relaxing the assumption that oseltamivir administration is restricted to severe influenza cases, by allowing between 0–5% of mild cases to be treated. One hundred combinations of values were chosen randomly from these ranges using Latin hypercube sampling, and simulations were run using each combination. The medians, interquartile ranges (IQR) and 95^th^ percentile ranges of model outcomes were then calculated across the simulations.

Since the aim of this study was to compare resource capacities across geographic areas and resource types, rather than to evaluate how transmission dynamics may vary across geographic areas, epidemiological parameters of the model were kept fixed in the multivariate uncertainty analysis to ensure comparability of resource capacity outcomes. However, due to the unpredictability of pandemic scenarios, we also explored model outcomes for a range of values for R_0_ and for the severe clinical attack rate in a separate univariate sensitivity analysis.

The model was coded and run in *R* version 2.10.1, using the “simecol” package [27] with the Runge-Kutta 4^th^ order algorithm for numerical integration of the differential equations. ArcGIS version 10 was used to map the calculated resource gaps and avoidable mortalities at provincial level.

## Results

### Resource gaps

**Figure 1:**
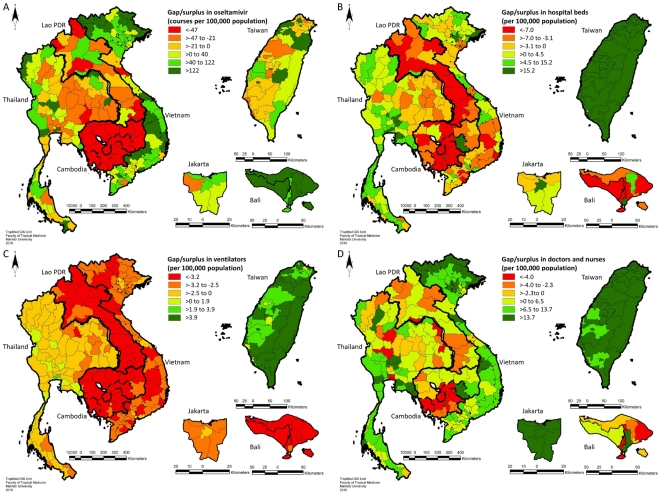
Geographical distribution of estimated health system resource gaps for a modeled pandemic influenza scenario. Gaps are mapped for oseltamivir (A), hospital beds (B), mechanical ventilators (C), and healthcare workers (pooled number of doctors and nurses; D). Areas shaded according to values ≤0 represent areas where a resource gap is predicted. Gaps are standardized by population size and mapped at province level for Cambodia, Lao PDR, Thailand, and Vietnam, at county level for Taiwan, and at district level for Jakarta and Bali in Indonesia.

Figure 1 presents the geographical distribution of estimated resource gaps across provinces (or districts in the case of Indonesia) in each study country for the modeled pandemic influenza scenario, under our baseline assumptions and point estimates for parameter values. The corresponding statistical distributions of resource capacities across areas within each country can be found in Figure S1. A summary of overall resource gaps for each country is presented in Table 1. There was substantial variation in resource gaps both between and within countries, and across resources types (Figures 1 and S1). Overall, the biggest gaps were generally seen in Cambodia and Lao PDR, particularly when standardized by population size, with almost all provinces in these countries displaying gaps in all resources, with the exception of nurses which were estimated to be sufficient in approximately half of the provinces in these countries. In contrast, relatively few provinces in Taiwan were estimated to have gaps, at least in general health system resources (beds, ventilators, and human resources), with quantities of these resources often considerably above those predicted to be needed for this scenario. Nevertheless, almost half of provinces in Taiwan were predicted to have insufficient oseltamivir supplies to treat all severe cases (although it should be noted that the results in Figures 1 and S1 do not account for central stockpiles which might be mobilized in the event of a pandemic, as discussed later).

**Table 1:** Summary of estimated resource gaps for a modeled pandemic influenza scenario.

	Cambodia	Indonesia*	Lao PDR	Thailand	Taiwan*	Vietnam
No. of provinces	24	15	17	76	25	63
Oseltamivir:						
No. (%) of provinces with a gap	24 (100)	1 (6.7)	13 (76.5)	40 (52.6)	11 (44.0)	5 (7.9)
Overall gap/surplus (excluding central stockpile)†	−9508.4	11449	−193.1	43746.5	13189.8	63964.5
Central stockpile†	31114	n/a‡	1445.6	513411	487000	n/a‡
Overall gap/surplus (including central stockpile)†	21605.6	11449.0‡	1252.5	557157.5	500190‡	63964.5
Hospital beds:						
No. (%) of provinces with a gap	21 (87.5)	10 (66.7)	16 (94.1)	24 (31.6)	1 (4.0)	32 (50.8)
Overall gap/surplus	−661.6	507.8	−365.3	3941	11031.2	1235.2
Ventilators:						
No. (%) of provinces with a gap	24 (100)	15 (100)	17 (100)	64 (84.2)	1 (4)	63 (100)
Overall gap/surplus	−440.4	−350.6	−190.1	−688.1	987.6	−2465.9
Doctors:						
No. (%) of provinces with a gap	21 (87.5)	5 (33.3)	15 (88.2)	67 (88.2)	3 (12)	2 (3.2)
Overall gap/surplus	−19.3	1504.4	−78.3	−1024.5	3682.5	2113.6
Nurses:						
No. (%) of provinces with a gap	12 (50)	5 (33.3)	10 (58.8)	8 (10.5)	2 (8)	1 (1.6)
Overall gap/surplus	139.9	1021.7	−44.7	3348.7	12277.4	6012.5

(Estimates are for comparative purposes only, and highly sensitive to model parameters and assumptions. They should not be interpreted as precise quantitative indications of resource gaps that may occur in a pandemic influenza scenario.)

*Data for Indonesia are from across districts (not provinces) in Jakarta and Bali only, and data for Taiwan are across counties.

† Values for oseltamivir represent the number of treatment courses.

‡ Data on central stockpiles of oseltamivir were not available for Indonesia and Vietnam.

Thailand, Indonesia and Vietnam generally displayed a more mixed picture. Results were comparable between Vietnam and Indonesia, with relatively few provinces of Vietnam (7·9%) and only one district of Jakarta (and none in Bali), estimated to have insufficient oseltamivir to treat all severe cases, with supplies of this antiviral drug comparably high across most other areas in these countries. Healthcare workers were also predicted to be mostly sufficient in Vietnam, with only 3·1% and 1·6% of provinces predicted to have a shortage of doctors and nurses, respectively, for this scenario. However, gaps in hospital beds were observed in over half of provinces in Vietnam and districts of Jakarta and Bali, and all of these provinces displayed a shortage of mechanical ventilators. Indeed, of all the resources, the largest gaps were observed in ventilators across all countries except Taiwan (Table 1).

Thailand had the second highest number of ventilators (absolute and per capita) after Taiwan, but a shortage of this resource was nevertheless predicted in over 80% of Thai provinces. A very heterogeneous pattern was observed in Thailand, with around 50% of provinces showing a shortage of hospital beds and oseltamivir, while many other provinces showed a clear “surplus” of the latter. Meanwhile, a shortage of medical doctors was predicted in over 80% of Thai provinces, with gaps in doctors comparable to those Lao PDR and Cambodia (Table 1). The number of nurses in Thai provinces was estimated to be somewhat more sufficient, however. A fairly distinct geographical pattern of resource gaps was evident in Thailand, particularly for oseltamivir and ventilators, with north-eastern (and some southern) provinces showing shortages more comparable with those in neighboring Lao PDR and Cambodia than with other Thai provinces (Figure 1).

It should be noted that estimates of gaps in beds, ventilators and human resources were highly dependent on the spare capacity of resources assumed to be available to care for pandemic influenza patients. For example, if spare capacity was assumed to be 5%, rather than 12%, then even in Taiwan most areas would suffer gaps in these resources for the modeled scenario. Furthermore, use of oseltamivir on even a fairly small proportion (5%) of mild cases, resulted in much faster depletion of this resource, such that all countries are predicted to experience a shortage of oseltamivir for treating all severe cases.

### Consequences of resource gaps on pandemic-associated mortality

**Figure 2:**
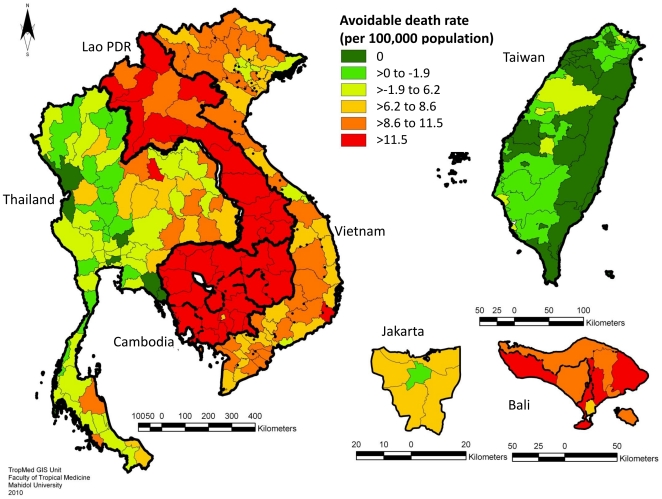
Geographical distribution of estimated avoidable mortality rates due to resource gaps for a modeled pandemic influenza scenario. Values are mapped at province level for Cambodia, Lao PDR, Thailand and Vietnam, at county level for Taiwan, and at district level for Jakarta and Bali in Indonesia.

**Figure 3:**
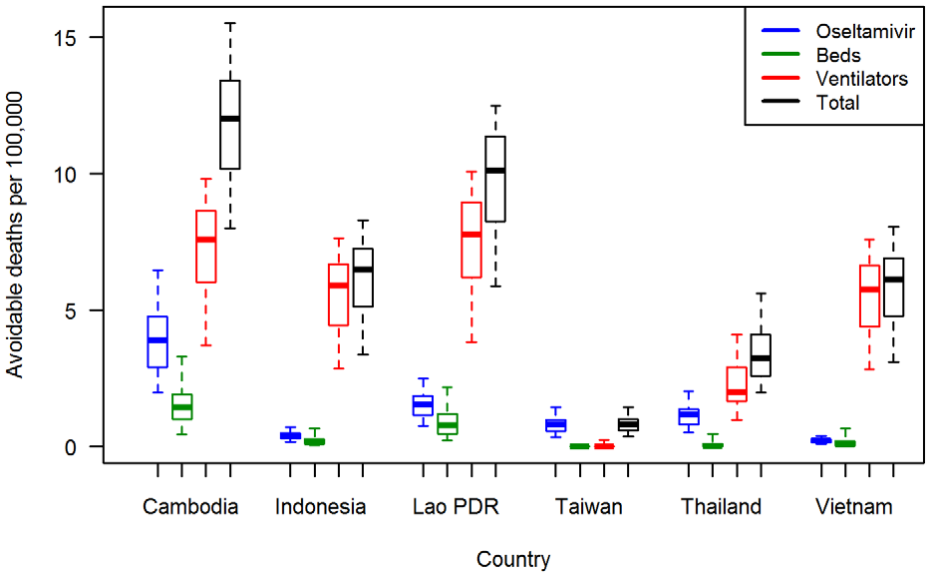
Estimated avoidable mortality rates due to each resource gap across countries for a modeled pandemic influenza scenario. Deaths attributed to shortages of oseltamivir, hospital beds, and ventilators are estimated from the number of deaths that the model predicts would be prevented by filling the gap in each of these resources alone. Boxplots show medians, interquartile ranges, and 95^th^ percentile ranges derived from a multivariate uncertainty analysis. Data are aggregated across provinces for Cambodia, Lao PDR, Thailand, Vietnam, and across counties for Taiwan. Data for Indonesia are aggregated across districts of Jakarta and Bali only.

The geographic distribution of avoidable deaths, estimated by calculating the number of deaths that would be prevented by filling all resource gaps in each province, and standardized by population size, is presented in Figure 2. (A corresponding map showing absolute numbers of estimated avoidable death is given in Figure S2.) Figure 3 shows estimated avoidable death rates attributable to gaps in each resource type (antivirals, beds and ventilators) aggregated across all provinces in each country, and accounting for uncertainty in resource effectiveness and spare capacity. Avoidable deaths for a given resource gap were estimated by calculating the number of deaths that would be prevented by filling that resource gap only.

Figures 2 and 3 highlight how resource gaps could have a substantial impact on mortality rates during an influenza pandemic. A combination of the large population size and shortage of ventilators results in the estimation that, out of the five countries for which nationwide data were collected, Vietnam would have the highest total number of avoidable deaths. However, the results for Jakarta and Bali suggest that Indonesia would have the highest avoidable death toll if the data is extrapolated across the entire population of this country. When standardized by population size, the highest rates of avoidable deaths were estimated in Cambodia and Lao PDR, accounting for over half of all pandemic-associated mortalities in these countries. The median avoidable death rates for these countries were over 15 times higher than that for Taiwan, where a relatively low proportion (median: 7.6%, IQR: 5.5–10.7%) of total deaths was estimated to be due to resource gaps. Almost all avoidable deaths in Taiwan were predicted to be due to local shortages of oseltamivir. In all other countries shortages of ventilators were estimated to be the biggest cause of avoidable deaths (Figure 3). In Indonesia, Lao PDR, and Vietnam, this result was largely robust to uncertainty surrounding resource effectiveness and spare capacity. For Cambodia and Thailand, however, the uncertainty analysis suggested that gaps in oseltamivir might also be a main cause of avoidable deaths (Figure 3). When adding an additional layer of uncertainty to the model assumptions, by allowing for up to 5% of mild cases to be treated with oseltamivir, the increased shortages of the latter further increased uncertainty surrounding the relative importance of gaps in oseltamivir (Figure S3). Given such uncertainties and the sensitivity of results to model assumptions, the proportion of mortalities that can be attributed to gaps in specific resources should be interpreted with some caution.

**Figure 4:**
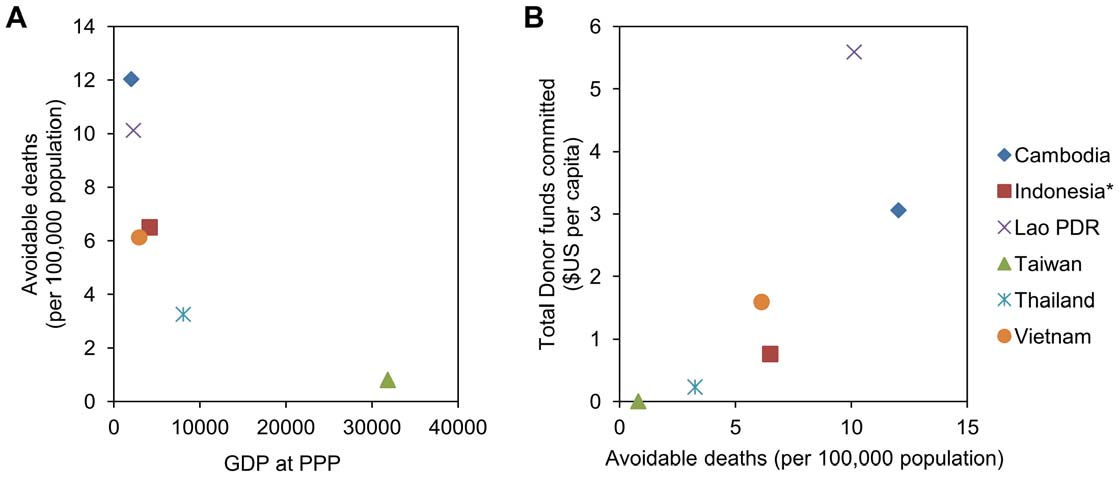
Association of predicted avoidable mortality rates with gross domestic product (A) and donor funding (B). Data on gross domestic product at product purchasing power (GDP at PPP) for 2009, obtained from the World Economic Outlook Database-April 2010, International Monetary Fund (http://www.imf.org/, accessed April 21^st^ 2010). Donor funds represent the total committed towards avian and human influenza up to December 2009, as reported in International Financial and Technical Assistance report for the International Ministerial Conference on Animal and Pandemic Influenza-2010, Hanoi, Vietnam (http://www.imcapi-hanoi-2010.org/documents/en/, accessed June 18^th^ 2010). *Avoidable mortality rates for Indonesia are estimated from Jakarta and Bali only.

A clear negative correlation was observed between estimated avoidable mortality rates and GDP per capita at country level (Figure 4A). Total funds per capita committed by donors towards avian and human influenza for each country, up to December 2009 [28] were positively correlated with avoidable mortality rates (Figure 4B).

### Resource distribution and mobilization

**Figure 5:**
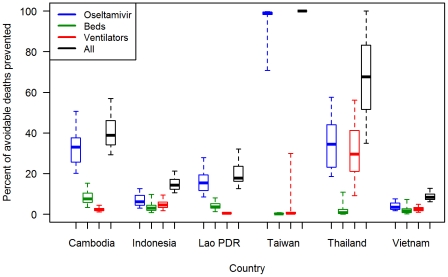
Estimated impact of resource mobilization/redistribution across provinces on avoidable mortality rates within each territory. Data were calculated by estimating the number of avoidable deaths if available resources (including central stockpiles for oseltamivir) within each territory were geographically distributed in proportion to provincial population size, and comparing with the total number of avoidable deaths predicted given actual resource distribution. Boxplots show medians, interquartile ranges, and 95^th^ percentile ranges derived from a multivariate uncertainty analysis. Data are aggregated across provinces for Cambodia, Lao PDR, Thailand, Vietnam, and across counties for Taiwan. Data for Indonesia are aggregated across districts of Jakarta and Bali only.

Within many countries it was evident that, while at least some provinces displayed resources gaps, other provinces were estimated to have more than sufficient resources for responding to the modeled scenario, with an overall “surplus” of some resources in several countries (Table 1). Furthermore, central stockpiles of oseltamivir were present in all countries from which data on this could be obtained. Thus we also investigated the proportion of avoidable deaths that might be averted in each country if the total available resources were equitably distributed across provinces according to provincial population size (Figure 5). When accounting for central stockpiles of oseltamivir, the overall supply of this drug was estimated to be sufficient to treat all severe cases in all countries (Table 1). Thus it was estimated that, in each country (except for Vietnam, and Jakarta and Bali, where provincial supplies of oseltamivir are already relatively high), effective mobilization of oseltamivir across administrative areas could potentially avert a significant proportion of the avoidable deaths estimated under current resource distributions (up to 100% of avoidable mortalities in Taiwan; Figure 5).

The (less feasible) scenario of redistributing available beds and ventilators according to provincial need within each country was generally estimated to have less of an impact on the number of avoidable deaths, compared to mobilization of oseltamivir (Figure 5). In the case of ventilators, this highlights how the large numbers of deaths attributed to gaps in this resource (Figure 3) are mostly due to overall nationwide shortages of ventilators, rather than an inequitable distribution of ventilators within most countries. In Thailand, however, if all ventilators were distributed in proportion to provincial population sizes, the model predicts around 30% (IQR: 21–41%) fewer avoidable deaths than the number predicted under the observed ventilator distribution.

### Sensitivity of results to pandemic severity

Estimates of resource gaps, and thus also avoidable mortalities, were very sensitive to the severity of the modeled pandemic scenario in relation to transmissibility and proportion of cases requiring hospitalization (Figure S4). A sensitivity analysis showed that under more severe (yet still plausible) pandemic scenarios, even Taiwan could experience substantial deaths due to shortages of hospital resources (Figure S4A and S4C). Furthermore, as the severity of the scenario increased, so too did the proportion of avoidable deaths that were attributable to gaps in hospital bed capacity (shown for Cambodia in Figure S4B and S4D). It is important to note, however, that for the ranges of values explored for the basic reproduction number and the proportion of cases that become severely ill, consistent patterns were observed when comparing relative magnitudes of avoidable mortality rates across countries (and also across provinces within countries).

## Discussion

Our results indicate that health system resource gaps for responding to a mild to moderate pandemic influenza scenario are wide and vary greatly, both within and between countries in Southeast Asia, and that these gaps could have a profound impact on pandemic-associated mortalities. Our estimates of resource gaps and avoidable mortality rates at country level show a clear association with national GDP. This result is consistent with a previous analysis of data from the 1918 influenza pandemic, which found that per capita income explained a large proportion of the variation in mortality across countries during the pandemic period [14]. Moreover, extrapolation of these mortality rates to the 2004 world population suggested that around 96% of deaths from pandemic influenza would occur in developing countries [14]. Our results suggest that, due to inequitable distribution of resources, the variation in pandemic burden is likely to be profound within, as well as between, countries.

Countries which have experienced the highest burden of Highly Pathogenic Avian Influenza (H5N1), namely Vietnam and Indonesia, appear to be most prepared in terms of the availability and geographical distribution of oseltamivir. In the other study countries, we estimated that central stockpiles of oseltamivir would be sufficient to cover any provincial gaps for treating all severe cases from the modeled scenario, and thus mobilization of this resource could potentially avert a large number of avoidable mortalities in these countries. Indeed, in all countries except Vietnam, we estimated that optimum mobilization of resources across administrative boundaries could save more than 10% of avoidable deaths. While timely mobilization of resources may be possible in Taiwan, with its small geographical size and relatively developed infrastructure, the feasibility of this scenario is questionable in the poorer, and larger, countries of the Mekong region, where it might be prudent to disburse central stockpiles of antiviral drugs to provincial and district health facilities prior to an outbreak.

Gaps in mechanical ventilators were predicted to be a major cause of avoidable deaths, with almost all provinces across all countries estimated to have severe shortages of this of this resource, with the exception of Taiwan and some Thai provinces. This pattern likely reflects the relatively high cost and human resource skills associated with acquisition and operation of ventilators, and highlights the importance of developing robust triage criteria as part of pandemic preparedness plans to ensure that this resource is allocated to the patients who are most likely benefit [29]–[30]. A previous analysis similarly suggested that a dire shortage of mechanical ventilators would be a major limiting factor in responding to a pandemic influenza outbreak in the United States [31]. We found particularly wide variation in the availability of ventilators, and indeed other hospital resources, in Thailand, where our results suggest that inequitable distribution of health system resources [32], rather than simply an overall nationwide shortage, could lead to a high number of avoidable deaths from pandemic influenza.

Of course, hospital equipment such as beds and ventilators are useless unless sufficient and qualified human resources are available to treat influenza patients, and our results suggest that gaps in healthcare workers would also be an important limiting factor for responding to pandemic influenza in many countries, particularly for Cambodia, Lao PDR and Thailand.

It is encouraging that total donor funds committed to avian and human influenza broadly correspond to avoidable mortality rates estimated at country level. A recent paper on Financial and Technical Assistance from the 2010 International Ministerial Conference on Animal and Pandemic Influenza (IMCAPI) reports that over 50% of total donor funding committed towards avian and human influenza worldwide between 2005 and 2009 was allocated towards “human health and pandemic preparedness” (with other funds committed towards sectors such as animal health; monitoring, information, and internal coordination; and information, education and communication) [28]. However, the extent to which these funds have been, or will be, allocated towards mitigating the resource gaps identified in our study is unknown to us and beyond the scope of this analysis.

This study is subject to several limitations, many of which relate to assumptions that were necessary for the modeled scenario. For example, in our baseline scenario we assumed that 12% of hospital capacity, across all provinces and countries, would be available to care for the surge of patients with influenza infections. In reality, surge capacity is likely to vary substantially between and within countries (and over time), but few data on this are available. Robust analytical frameworks are urgently needed to define and measure health system surge capacity in order to inform analyses of resource gaps for emergency response scenarios. The effectiveness of resources such as antiviral drugs, ventilators, and general hospital care for improving the survival rates among severe influenza cases is also surrounded by considerable uncertainty and may vary between settings. Our results show that, even if epidemiological parameters describing transmission and pathogenicity are kept constant, uncertainties in spare capacity and resource effectiveness lead to considerable uncertainties in estimates of avoidable mortalities rates. Nevertheless, the distributions of model outputs from our multivariate uncertainty analysis still showed some significant differences when compared across countries and across resource types (Figures 3 and 5). Furthermore, it seems likely that spare capacity and resource effectiveness would be higher in more resource-rich settings, which would only strengthen the findings of this study in terms of the geographic distribution of resource gaps and avoidable mortalities.

Estimates of resource gaps and avoidable mortality rates were also very sensitive to parameters describing pandemic severity, although similar patterns were observed across geographic areas for the range of values explored. However, the same cannot be said for the relative importance of gaps in different resource types. Thus, although our results generally suggest that shortages of ventilators could be a major cause of avoidable deaths in low- and middle-income countries in Southeast Asia, investment in this resource should not necessarily be prioritized over other healthcare resources. A natural extension of this study would be to investigate the cost-effectiveness of investing different types of health systems resources for mitigating the burden of an influenza pandemic. However, more data on the effectiveness of different resources for managing severe influenza cases is needed before such assessments can be made.

Other limitations relate to the simplicity of the model structure. We assumed homogenous mixing and a constant basic reproduction number across all populations. In reality, heterogeneities in factors such as age-structure, geographic structure, population density, human behavior, and the underlying health of the population are all likely to play a role in transmission dynamics and burden of influenza. A previous modeling analysis, for example, has shown that higher levels of population heterogeneities, such as in age and spatial structuring of contacts, result in lower overall attack rates and peak prevalence for a given basic reproduction number [33]. However, there is a lack of data on such heterogeneities and how they might affect patterns of pandemic progression for our study region. Ongoing studies, such as contact pattern surveys in Asia similar to those undertaken in Europe [34], are attempting to rectify this. Another limitation is that the resource data were collected between May and September 2009, which includes the first wave of the H1N1-2009 pandemic; thus some resource data (particularly for antiviral stockpiles) may be influenced by the time point within this period at which the data were recorded.

Given the above caveats, it is important to emphasize that we do not advocate these results to be accurate quantitative reflections of resources shortages or deaths that are likely to occur in any given pandemic scenario. Rather, they highlight the scale of health system inequalities within and across countries in Asia, and the considerable impact such inequalities could have on the pandemic disease burden. By indicating the relative disparities in resource availability within and across countries, and the potential consequences of resource shortages, these results could help guide investment decisions in scaling up resources to mitigate the burden of future pandemics. As many of these resources have a generic healthcare function beyond pandemic influenza, they may also be useful to guide health system strengthening.

## Supporting Information

Figure S1 **Variation in estimated resource capacities across provinces within each territory for the modeled pandemic scenario.**
(DOCX)

Figure S2 **Geographical distribution of estimated avoidable deaths due to resource gaps for a modeled pandemic influenza scenario.**
(DOCX)

Figure S3 **Estimated avoidable mortality rates by resource gap when oseltamivir usage is not restricted to severe influenza cases.**
(DOCX)

Figure S4 **Sensitivity of model outputs to changes in pandemic severity.**
(DOCX)

Text S1 **Mathematical model structure and assumptions.**
(DOCX)

Table S1 **Model parameters and values.**
(DOCX)
