# Supplementary material for: Health System Resource Gaps and Associated Mortality from Pandemic Influenza across Six Asian Territories
Source: PLoS One. 2012 Feb 21;7(2):e31800. doi: 10.1371/journal.pone.0031800 (PMC3283680; doi:10.1371/journal.pone.0031800)
Supplement: Figure S1 — Variation in estimated resource capacities across provinces within each territory for the modeled pandemic scenario. (DOCX) [file pone.0031800.s001.docx]

**Figure S1.** **Estimated resource capacities across provinces within each territory** **for a modeled pandemic scenario.** Gaps/surpluses are estimated for oseltamivir (A and B), hospital beds (C and D), mechanical ventilators (E and F), medical doctors (G and H) and nurses (I and J). Values ≤ 0 indicate resource gaps. Plots on the left represent absolute numbers, plots on the right represent values standardised by population size. The boxes depict medians and interquartile ranges (IQR), and whiskers depict minimum and maximum values within 1.5 IQR of the lower and upper quartiles, respectively (values outside this range are shown as outliers). Values for Indonesia represent data across districts for Jakarta and Bali only. Values for Taiwan represent data across counties. All other data are across provinces in each country. cm = Cambodia, id = Indonesia, la = Lao PDR, th = Thailand, tw = Taiwan, vn = Vietnam.
