# Supplementary material for: Health System Resource Gaps and Associated Mortality from Pandemic Influenza across Six Asian Territories
Source: PLoS One. 2012 Feb 21;7(2):e31800. doi: 10.1371/journal.pone.0031800 (PMC3283680; doi:10.1371/journal.pone.0031800)
Supplement: Figure S2 — Geographical distribution of estimated avoidable deaths due to resource gaps for a modeled pandemic influenza scenario. (DOCX) [file pone.0031800.s002.docx]

**Figure S2.** **Geographical distribution of estimated avoidable deaths due to resource gaps for a modeled pandemic influenza scenario**. Values are mapped at province level for Cambodia, Lao PDR, Thailand and Vietnam, at county level for Taiwan, and at district level for Jakarta and Bali in Indonesia.
