# Supplementary material for: Health System Resource Gaps and Associated Mortality from Pandemic Influenza across Six Asian Territories
Source: PLoS One. 2012 Feb 21;7(2):e31800. doi: 10.1371/journal.pone.0031800 (PMC3283680; doi:10.1371/journal.pone.0031800)

**Figure S3. Estimated avoidable mortality rates by resource gap when oseltamivir usage is not restricted to severe influenza cases.** As Figure 3, but allowing for between 0-5% of mild influenza cases to be treated with oseltamivir (in addition to treatment of severe cases) in the multivariate uncertainty analysis.


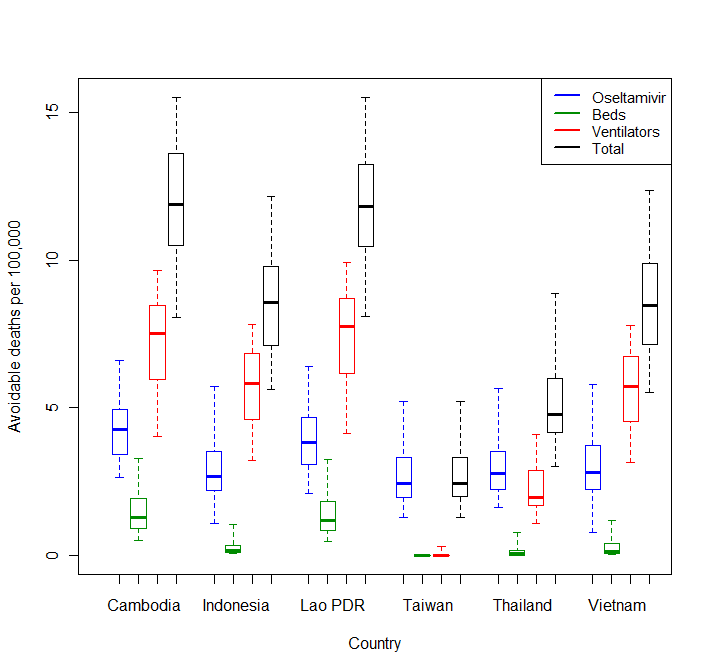

Supplement: Figure S3 — Estimated avoidable mortality rates by resource gap when oseltamivir usage is not restricted to severe influenza cases. (DOCX) [file pone.0031800.s003.docx]
