# Supplementary material for: Health System Resource Gaps and Associated Mortality from Pandemic Influenza across Six Asian Territories
Source: PLoS One. 2012 Feb 21;7(2):e31800. doi: 10.1371/journal.pone.0031800 (PMC3283680; doi:10.1371/journal.pone.0031800)
Supplement: Figure S4 — Sensitivity of model outputs to changes in pandemic severity. (DOCX) [file pone.0031800.s004.docx]

**Figure S4. Sensitivity of model outputs to changes in pandemic severity.** Plots show the change in avoidable mortality rates with respect to changes in the basic reproduction number (A and B), and clinical severity (C and D). Plots on the left show avoidable mortality rates by country. Plots on the right show avoidable mortality rates attributed to gaps in each resource type, as estimated for Cambodia (which displayed the largest gaps overall).
