# Supplementary material for: Health System Resource Gaps and Associated Mortality from Pandemic Influenza across Six Asian Territories
Source: PLoS One. 2012 Feb 21;7(2):e31800. doi: 10.1371/journal.pone.0031800 (PMC3283680; doi:10.1371/journal.pone.0031800)
Supplement: Text S1 — Mathematical model structure and assumptions. (DOCX) [file pone.0031800.s005.docx]

**Text S1. Mathematical Model Structure and Assumptions**

The model structure, detailed fully in Krumkamp *et al.* [1], is based on a Susceptible-Exposed-Infectious-Removed (SEIR) model. The population was divided into 17 compartments, defined as Susceptible (S); Exposed (E); Pre-symptomatic infectious cases (A); Asymptomatic infection (I_a_ and I_a2_); Mild infection (I_m_); Mild infection with antiviral treatment (I_ma_), mild infection without antiviral treatment (I_m2_); Critically ill requiring hospitalization (I_c_); Hospitalized and infectious (I_h_); Hospitalized and non-infectious (I_h2_); Critically ill at home with antiviral treatment (I_ca_); Critically ill at home without antiviral treatment (I_c2_); Ventilated and infectious (V); Ventilated and non-infectious (V_2_); Dead (D); and Recovered (R).

The differential equations of the model, defining the rates of change in these state variables over time, are defined as follows:

$\begin{matrix} \frac{dS}{dt}=-q\kappa(1-\alpha p_{\kappa})\frac{S}{N-D}(A+I_{a}+I_{a2}+I_{m}+I_{m2}+I_{ma}+I_{c}+I_{c2}+I_{ca})- \\ q(1-p_{qh})\kappa_{h}\frac{S}{N-D}(I_{h}+V) \end{matrix}$ (1)

$\begin{matrix} \frac{dE}{dt}=q\kappa(1-\alpha p_{\kappa})\frac{S}{N-D}(A+I_{a}+I_{a2}+I_{m}+I_{m2}+I_{ma}+I_{c}+I_{c2}+I_{ca})+ \\ q(1-p_{qh})\kappa_{h}\frac{S}{N-D}(I_{h}+V)-\sigma E \end{matrix}$ (2)

$\frac{dA}{dt}=\sigma E-\delta A$ (3)

$\frac{dI_{a}}{dt}=\delta p_{a}A-\tau I_{a}$ (4)

$\frac{dI_{a2}}{dt}=\tau I_{a}-\gamma_{a}I_{a2}$ (5)

$\frac{dI_{m}}{dt}=\delta p_{m}A-\tau I_{m}$ (6)

$\frac{dI_{ma}}{dt}=\Theta(\varepsilon)p_{ma}\tau I_{m}-\gamma_{ma}I_{ma}$ (7)

$\frac{dI_{m2}}{dt}=\Theta(\varepsilon)(1-p_{ma})\tau I_{m}+(1-\Theta(\varepsilon))\tau I_{m}-\gamma_{m}I_{m2}$ (8)

$\frac{dI_{c}}{dt}=\delta p_{c}A-\tau I_{c}$ (9)

$\frac{dI_{h}}{dt}=\Theta(\phi)(1-p_{v})\tau I_{c}-\Theta(\varepsilon)\gamma_{ca}I_{h}-(1-\Theta(\varepsilon))\gamma_{c}I_{h}$ (10)

$\frac{dI_{h2}}{dt}=\Theta(\varepsilon)\gamma_{ca}I_{h}+(1-\Theta(\varepsilon))\gamma_{c}I_{h}-\gamma_{h}I_{h2}$ (11)

$\frac{dI_{ca}}{dt}=\Theta(\varepsilon)(1-\Theta(\phi))(1-p_{v})\tau I_{c}-\gamma_{ca}I_{ca}$ (12)

$\frac{dI_{c2}}{dt}=(1-\Theta(\varepsilon))(1-\Theta(\phi))(1-p_{v})\tau I_{c}-\gamma_{c}I_{c2}$ (13)

$\frac{dV}{dt}=\Theta(\omega)p_{v}\tau I_{c}-\Theta(\varepsilon)\gamma_{ca}V-(1-\Theta(\varepsilon))\gamma_{c}V$ (14)

$\frac{dV_{2}}{dt}=\Theta(\varepsilon)\gamma_{ca}V+(1-\Theta(\varepsilon))\gamma_{c}V-\gamma_{v}V_{2}$ (15)

$\frac{dD}{dt}=\gamma_{c}d_{c}I_{c2}+\gamma_{ca}d_{ca}I_{ca}+\Theta(\varepsilon)\gamma_{h}d_{ha}I_{h2}+(1-\Theta(\varepsilon))\gamma_{h}d_{h}I_{h2}+\gamma_{v}d_{v}V_{2}+(1-\Theta(\omega))p_{v}\tau I_{c}$ (16)

$\begin{matrix} \frac{dR}{dt}=\rho vS+\gamma_{a}I_{a2}+\gamma_{m}I_{m2}+\gamma_{ma}I_{ma}+\gamma_{c}(1-d_{c})I_{c2}+\gamma_{ca}(1-d_{ca})I_{ca}+\Theta(\varepsilon)\gamma_{h}(1-d_{ha})I_{h2}+ \\ (1-\Theta(\varepsilon))\gamma_{h}(1-d_{h})I_{h2}+\gamma_{v}(1-d_{v})V_{2} \end{matrix}$(17)

Parameter values and their sources are shown in Table S1. Susceptible individuals (S) become infected at a force of infection dependent upon the per capita contact rate (k), the probability of transmission upon contact with an infectious person (q), and the proportion of individuals within the total population that are infectious. In this scenario it was also assumed that the contact rate within the population would be reduced during periods when over 0.5% of the population is symptomatically infected. This was based on the assumption that, during periods of high pandemic activity, individuals will change their behaviour to reduce contacts, and/or social distancing measures such as school closure will be implemented.

Newly infected individuals move sequentially through an exposed (but not yet symptomatic or infectious) compartment (E), followed by a an infectious, asymptomatic period (A). After this, a proportion (p­_a_) of those infected will will remain asymptomatic, moving sequentially to compartments I_a_ and I_a2_. Another proportion of infected individuals (*p*_m_) will develop mild symptoms and move to compartment I_m_, while the remaining proportion, *p*_c_, will become critical cases with severe infections (I_c_). In the base-case modelled scenario we assumed that 30 %, 69.8 %, and 0.2% of cases will be asymptomatic, mild and critical, respectively, and that these case groups differ from eachother in their duration of infectiousness.

We assumed that all critical cases will require antiviral treatment and hospitalisation, and that a proportion (*p*_v_) require mechanical ventilation, although whether they receive these depended upon the availabitilty of oseltamivir, hospital beds, and ventilators, defined by parameters ε, ω, and φ repctively, which take a value of 1 if the resource is available, and 0 if the resource is depleted (in the case of oseltamivir) or fully occupied (in the case of beds and ventilators). If ventilators are available, those cases requiring ventilation would move to the ventilated group (V_1_), otherwise they are assumed to die. If hospital beds are available, other critical cases will become hospitalised and move move to compartment I_h_, otherwise they become critical outpatients with or without antiviral treament (I_ca_ and I_c2_, respectively), depending on the availability of oseltamivir. For these groups different death rates and different infectious periods apply according to the severity and whether they receive the healthcare resources they require. Hospitalised and ventilated cases are assumed to remain in hospital for a period after their infectiousness has disappeared, as denoted by compartments I_h2_ and I_v2_, respectively.

If oseltamivir is available, a proportion of mild cases (p_ma_) could also receive antiviral treatment and move to compartment I_ma_, although this was set to zero in our scenario, under the assumption that only severe cases would be treated. All asymptomatic and mild cases were assumed to recover from infection.

***Depletion of health system resources***

The number of oseltamivir courses available within a province was assumed to be depleted at a rate of one course per new severe infection, with one course defined as ten capsules (75 mg twice daily for five days).

For general health system resources, we assumed that 12 % of the total number of hospital beds, ventilators, doctors and nurses, as recorded from the resource survey data, would be available to care for pandemic influenza cases. Therefore, the number of available beds and ventilators at time *t* can be defined as:

$$N_{BedsAvailable}(t)=0.12*N_{BedsTotal}-(I_{h}(t)+I_{h2}(t))$$

$$N_{VentilatorsAvailable}(t)=0.12*N_{VentilatorsTotal}-(V(t)+V_{2}(t))$$

The overall numbers of beds and ventilators needed were calculated from the peak number of hospitalised and ventilated cases, respectively, predicted from a simulation of the model with unlimited resources. The peak number of hospitalised cases from this simulation was also used to estimate the needed number of doctors and nurses. For this, we assumed that the healthcare workforce was divided into two day shifts and one night shift. During a day and night shift, we assumed that one nurse can care for up to five and ten cases, and one physician can care for ten and 40 cases, respectively.

**References**

1. Krumkamp R, Kretzschmar M, Rudge JW, Ahmad A, Hanvoravongchai P, et al. (2011) Health service resource needs for pandemic influenza in developing countries: a linked transmission dynamics, interventions and resource demand model. Epidemiology and Infection 139: 59-67.
