# Supplementary material for: Health System Resource Gaps and Associated Mortality from Pandemic Influenza across Six Asian Territories
Source: PLoS One. 2012 Feb 21;7(2):e31800. doi: 10.1371/journal.pone.0031800 (PMC3283680; doi:10.1371/journal.pone.0031800)
Supplement: Table S1 — Model parameters and values. (DOCX) [file pone.0031800.s006.docx]

**Table S1.** Model parameters and values

| **Parameter** | | **Description** | **Value** | | **Justification/ Notes** | | |  |
| --- | --- | --- | --- | --- | --- | --- | --- | --- |
| к | | Rate of daily contacts possibly resulting in transmission | 7 | | Assumption for the model, based on R_0_ (к=R_0_γ/q) | | |  |
| q | | Proportion of contacts resulting in transmission | 0.09 | | Assumption based on [1] | | |  |
| σ | | Rate at which individuals leave exposed group | 1 | | 1-2 days [1], 2.62 days [2], and 1-5 days [3] between infection and symptom onset | | |  |
| δ | | Rate at which individuals leave asymptomatic group | 1/0.4 | | 1 day [2-3], and 0-2 days [3] infectious before symptom onset | | |  |
| τ | | Rate cases get detected | 2 | | Assumption for the model | | |  |
| p_κ_ | Proportion of contacts reduced during contact reduction period (when prevalence of symptomatic cases > 0.5%) | | | 0.1 | | | Assumption for the model | |
| p_qh_ | | Proportion of hospital transmissions reduced (e.g. due to isolation) | 0.7 | | Assumption for the model | | |  |
| p_a_ | | Proportion of cases that are asymptomatic | 0.3 | | values range from 30 to 50% [1,4-6] | | |  |
| p_m_ | | Proportion of cases that are mild | 0.698 | | p_m_=1-p_a_-p_c_ | | |  |
| p_c_ | | proportion of cases that are critical (require hospitalisation) | 0.002 | | 0.16% [7] and 0.45% [8] of symptomatic cases hospitalised | | |  |
| p_v_ | | Proportion of hospitalised cases needing ventilation | 0.2 | | 21% of hospitalised cases needing intensive care treatment [9], 18% of hospitalised cases needing ventilation [10-11], 0.002 to 0.035% of symptomatic cases need ICU treatment | | |  |
| γ_a_ | | Rate at which asymptomatic cases leave infectious group | 2 | | 1.9 days [12], 3.38 days [2], 4-10 days [3] mean duration of infectivity | | |  |
| γ_m_ | | Rate at which mild cases leave infectious group | 1/1.5 | | See above [2-3,12] | | |  |
| γ_c_ | | Rate at which severe cases leave infected group | 1/3.5 | | See above [2-3,12] | | |  |
| γ_ma_  γ_ca_ | | Rate at which mild and critical cases under AV treatment leave infectious groups | 2  1/2.5 | | Infectious period under AV treatment reduced by 1 day [4] | | |  |
| γ_h_ | | Rate at which cases leave hospital group | 1/12 | | Length of ICU stay 13.5 days [13] | | |  |
| γ_v_ | | Rate at which cases leave ventilated group | 1/13 | | 12 days [14], 15 days [13] | | |  |
| d_c_ | | Proportion of critical outpatients without AV treatment that die | 0.25 | | Proportions of deaths are extrapolated from the following data: 40% of ICU cases died [13], 14.3% of ICU cases died [14], 11% of hospitalised cases died [11], 7 % of hospitalised cases died [15], 0.007% of symptomatic cases died [7] | | |  |
| d_ca_ | | Proportion of critical outpatients under AV treatment that die | 0.15 | | Based on d_ca_ = d_c_(1-π_a_) | | |  |
| d_h_ | | Proportion of hospitalised cases without AV treatment that die | 0.10 | | Based on d_h_ = d_c_(1-π_h_) | | |  |
| d_ha_ | | Proportion of hospitalised cases under AV treatment that die | 0.06 | | Based on d_ha_ = d_c_(1-π_a_)(1-π_h_) | | |  |
| d_v_ | | Proportion of ventilated cases that die | 0.25 | | Based on d_v_ = (1- π_v_) | | |  |
| π_a_ | | Effectiveness of antiviral treatment at reducing death rate in critical cases | 0.4 | | Assumption based on [7,13-14], Odds Ratio for reduction in influenza mortality in hospitalised cases: 0.21 (95% confidence interval 0.06-0.80) [16], patients who died were less likely to have received AV therapy within 48 hours after symptom onset [15]. (Varied between 0.2-0.8 in uncertainty analysis.) | | |  |
| π_h_ | | Effectiveness of hospitalised care (without AV treatment) at reducing death rate in critical cases | 0.6 | | Assumption based on [7,13-14]. (Varied between 0.2-0.8 in uncertainty analysis.) | | |  |
| π_v_ | | Effectiveness of ventilators at preventing death in cases needing ventilation | 0.75 | | Assumption based on [7,13-14], 45% of ventilated cases died [15]. (Varied between 0.2-0.8 in uncertainty analysis.) | | |  |
| p_ma_ | | Proportion of mild cases treated with oseltamivir | 0 | | In this scenario we assumed only critical cases are treated with oseltamivir. (Varied between 0 - 0.05 in uncertainty analysis) | | |  |
| **Parameters describing resource availability:** | | | | | | | |  |
| ε | | Availability of oseltamivir drugs: | | | | $\Theta(\varepsilon)=\left\{\begin{matrix} 1,N_{OselAvailable}(t)>0 \\ \text{0, }N_{OselAvailable}(t)=0 \end{matrix}\right.$ | |  |
| φ | | Availability of hospital beds: | | | | $\Theta(\varphi)=\left\{\begin{matrix} \text{1, }N_{BedsAvailable}(t)>0 \\ \text{0, }N_{BedsAvailable}(t)=0 \end{matrix}\right.$ | |  |
| ω | | Availability of medical ventilators: | | | | $\Theta(\omega)=\left\{\begin{matrix} \text{1, }N_{VentilatorsAvailable}(t)>0 \\ \text{0, }N_{VentilatorsAvailable}(t)=0 \end{matrix}\right.$ | |  |

**References**

1. Longini IM, Jr., Nizam A, Xu S, Ungchusak K, Hanshaoworakul W, et al. (2005) Containing pandemic influenza at the source. Science 309: 1083-1087.

2. Tuite AR, Greer AL, Whelan M, Winter AL, Lee B, et al. (2009) Estimated epidemiologic parameters and morbidity associated with pandemic H1N1 influenza. CMAJ.

3. Pourbohloul B, Ahued A, Davoudi B, Meza R, Meyers LA, et al. (2009) Initial human transmission dynamics of the pandemic (H1N1) 2009 virus in North America. Influenza Other Respi Viruses 3: 215-222.

4. Fraser C, Riley S, Anderson RM, Ferguson NM (2004) Factors that make an infectious disease outbreak controllable. Proc Natl Acad Sci U S A 101: 6146-6151.

5. Longini IM, Jr., Halloran ME, Nizam A, Yang Y (2004) Containing pandemic influenza with antiviral agents. Am J Epidemiol 159: 623-633.

6. Duerr HP, Brockmann SO, Piechotowski I, Schwehm M, Eichner M (2007) Influenza pandemic intervention planning using InfluSim: pharmaceutical and non- pharmaceutical interventions. BMC Infect Dis 7: 76.

7. Presanis AM (2009) The Severity of Pandemic H1N1 Influenza in the United States, from Apil to July 2009: A Bayesian Analysis. PLOS Medicine 6: e1000207.

8. Reed C, Angulo FJ, Swerdlow DL, Lipsitch M, Meltzer MI, et al. (2009) Estimates of the prevalence of pandemic (H1N1) 2009, United States, April-July 2009. Emerg Infect Dis 15: 2004-2007.

9. Siva N (2009) Number of swine flu patients going into intensive care is rising. BMJ 339: b4528.

10. Ercole A, Taylor BL, Rhodes A, Menon DK (2009) Modelling the impact of an influenza A/H1N1 pandemic on critical care demand from early pathogenicity data: the case for sentinel reporting. Anaesthesia 64: 937-941.

11. Louie JK, Acosta M, Winter K, Jean C, Gavali S, et al. (2009) Factors associated with death or hospitalization due to pandemic 2009 influenza A(H1N1) infection in California. JAMA 302: 1896-1902.

12. Flahault A, Vergu E, Boelle PY (2009) Potential for a global dynamic of Influenza A (H1N1). BMC Infect Dis 9: 129.

13. Dominguez-Cherit G, Lapinsky SE, Macias AE, Pinto R, Espinosa-Perez L, et al. (2009) Critically Ill patients with 2009 influenza A(H1N1) in Mexico. JAMA 302: 1880-1887.

14. Kumar A, Zarychanski R, Pinto R, Cook DJ, Marshall J, et al. (2009) Critically ill patients with 2009 influenza A(H1N1) infection in Canada. JAMA 302: 1872-1879.

15. Jain S, Kamimoto L, Bramley AM, Schmitz AM, Benoit SR, et al. (2009) Hospitalized patients with 2009 H1N1 influenza in the United States, April-June 2009. N Engl J Med 361: 1935-1944.

16. McGeer A, Green KA, Plevneshi A, Shigayeva A, Siddiqi N, et al. (2007) Antiviral therapy and outcomes of influenza requiring hospitalization in Ontario, Canada. Clin Infect Dis 45: 1568-1575.
